# In Vitro Effects of Triamcinolone and Methylprednisolone on the Viability and Mechanics of Native Articular Cartilage

**DOI:** 10.1177/03635465231162644

**Published:** 2023-07-16

**Authors:** Nathan P. Fackler, Evelia Yareli-Salinas, Kylie T. Callan, Kyriacos A. Athanasiou, Dean Wang

**Affiliations:** * Department of Orthopaedic Surgery, University of California, Irvine, Orange, California, USA; † Department of Biomedical Engineering, University of California, Irvine, Irvine, California, USA; Investigation performed at the University of California Irvine, Irvine, California, USA

**Keywords:** injection, steroid, methylprednisolone, triamcinolone, articular cartilage, mechanics

## Abstract

**Background:**

The chondrotoxic effects of methylprednisolone acetate (MP) and triamcinolone acetonide (TA) have been well described. However, the mechanical effects of these commonly used steroids on native cartilage are largely unknown.

**Purpose:**

To investigate the in vitro effects of a single 1-hour MP or TA exposure on the viability, mechanics, and biochemical content of native articular cartilage explants.

**Study Design:**

Controlled laboratory study.

**Methods:**

Articular cartilage explants (n = 6 per group) were harvested from the femoral condyles of bovine stifles. Explants were exposed to chondrogenic medium containing a clinical dose of MP or TA for 1 hour, followed by fresh medium wash and exchange. Explants in the control group underwent the same treatment with chondrogenic medium alone. At 24 hours after treatment, samples were assessed for viability (live/dead), mechanical properties (creep indentation and Instron tensile testing), biochemical (collagen and glycosaminoglycan) content, and pyridinoline crosslinking via mass spectrometry.

**Results:**

Mean cell viability was significantly decreased in native explants exposed to MP (35.5%) compared with the control (49.8%; *P* < .001) and TA (45.7%; *P* = .01) specimens. Significant decreases were seen in the mechanical properties of steroid-treated native explants when compared with controls, with decreases in aggregate modulus (646.3 vs 312.8 kPa [MP] and 257.0 kPa [TA]; *P* < .001), shear modulus (370.1 vs 191.2 kPa [MP] and 157.4 kPa [TA]; *P* < .001), and ultimate tensile strength (9.650 vs 5.648 MPa [MP; *P* = .021] and 6.065 MPa [TA; *P* = .0403]). No significant differences in collagen and glycosaminoglycan content were found in the steroid-treated groups. Pyridinoline crosslinking was significantly decreased in explants exposed to TA compared with controls (*P* = .027).

**Conclusion:**

Exposure of MP to articular cartilage explants was chondrotoxic, and exposure of articular cartilage explants to MP or TA resulted in significant decreases in mechanical properties of articular cartilage explants compared with controls. Clinicians should be judicious regarding use of intra-articular steroids, particularly in patients with intact healthy articular cartilage.

Osteoarthritis continues to be a prevalent issue causing joint pain and dysfunction, with knee osteoarthritis affecting >9 million adults and costing the health care system $27 billion annually in the United States.^25^ Intra-articular (IA) injection of corticosteroids is commonly performed for the nonoperative management of joint arthritis.^34^ Two of the most commonly used corticosteroids for therapeutic IA injection are methylprednisolone acetate (MP) and triamcinolone acetonide (TA).^36^ These 2 steroids have been shown to provide pain relief after a single injection, with higher doses leading to relatively longer lasting pain reduction and functional improvement.^31^

The appropriate use of IA corticosteroids has remained controversial among clinicians due to recent basic science studies and clinical controlled trials demonstrating a potential chondrotoxic effect of MP and TA on articular cartilage.^18,36^ Although MP has been found to be protective in low, subtherapeutic doses by preventing cartilage degeneration through the suppression of matrix metalloproteinases and urokinase plasminogen activator,^8^ doses >7 mg MP can lead to significant chondrotoxicity. These doses have additionally been correlated with changes in cartilage thickness, fibrillation, and inhibition of cell maturation.^36^ Basic science data on TA have demonstrated a chondrotoxic effect at all doses.^36^ However, clinical studies on TA have shown mixed results, with some trials showing prevention of joint space narrowing on radiographs,^26^ others showing loss of cartilage volume on magnetic resonance imaging,^18^ and others showing no change to the cartilage but decreases in meniscal volume.^22^

The biochemical and biomechanical properties of articular cartilage have also been shown to change with exposure to MP and TA, although these effects have not been well studied. IA injections of MP have been correlated with decreases in glycosaminoglycan (GAG) content with detrimental changes to the cartilage mechanical properties, including changes in compressive stiffness, permeability, shear modulus, and thickness, perhaps predisposing the cartilage to mechanical failure.^15,19^ Injections of TA have been correlated with similar decreases in both GAG and collagen content.^3,37^ However, no studies to date have examined the effect of TA exposure on the mechanical properties of articular cartilage.

Due to the prevalence of these IA injections in current clinical practice and the uncertainties regarding their effects on the extracellular matrix of healthy articular cartilage, further investigation into their potential effects is warranted. The purpose of this study was to evaluate the effects of a single MP or TA exposure on the viability, biochemistry, and biomechanics of articular cartilage using bovine explants. The hypothesis was that a single corticosteroid exposure would lead to decreases in GAG and collagen content, corresponding to decreases in the mechanical properties of articular cartilage explants.

## Methods

### Explant Harvest

Full-thickness (approximately 3-4 mm) cartilage explants were harvested from the femoral condyles of 6 juvenile (6-month-old) bovine stifle joints (Research 87 Inc) 36 hours after slaughter using a 5 mm–diameter punch knife under antiseptic technique. Bovine articular cartilage has been validated as an appropriate model for cartilage pathology due to its homology to human articular cartilage in both structure and viscoelastic properties.^33^ Explants were rinsed in Dulbecco's modified Eagle medium (DMEM; Gibco) with high glucose/GlutaMAX and 1% (vol/vol) penicillin/streptomycin/fungizone (PSF; BD Biosciences). Explants were maintained in chondrogenic medium (DMEM with high glucose/GlutaMAX containing 1% PSF, 1% [vol/vol] non–essential amino acids, 100 nM dexamethasone, 40 µg/mL L-proline, 50 µg/mL ascorbate-2-phosphate, and 100 mg/mL sodium pyruvate; all from Sigma) and 3% (vol/vol) fetal bovine serum (Atlanta Biologicals) until steroid exposure later that day.

### Steroid Exposure

Native explants were exposed to chondrogenic medium supplemented with MP (40 mg/mL), TA (40 mg/mL), or nothing (negative control) (n = 6 explants per group) for 1 hour at 37°C using an orbital shaker. Steroid doses were calculated based on a standard clinical 1-mL bolus injection into an adult human knee while accounting for a dilution of 6.7 mL of synovial joint fluid.^13^ For example, a 1-mL (40 mg) bolus of TA injected into a knee joint with 6.7 mL of synovial fluid yields 5.97 mg/mL within the joint. Using this calculated concentration, we placed native explants in a 24-well plate and incubated them with 1.2 mL of appropriate steroid-chondrogenic medium dilution to allow for complete submersion of the tissues. After the 1-hour exposure, explants were washed 3 times with chondrogenic medium. Explants were then maintained in chondrogenic medium overnight at 37°C and 10% CO_2_ until testing at 24 hours after steroid exposure. Each 5 mm–diameter explant was partitioned into 5 individual specimens for viability, biochemistry, biomechanical, and histological analyses according to established protocols,^11,21,38^ allowing n = 6 for all analyses in this study.

### Viability Assessment

Vertical cross sections, approximately 1 mm thick, were prepared and incubated in 80 µL of chondrogenic medium plus 80 µL of LIVE/DEAD reagent (calcein acetoxymethyl, ethidium homodimer-1; Thermo Fisher) for 30 minutes. Sections were viewed via fluorescence microscopy using the Texas red and green fluorescent filters at ×4 and ×20 magnification. Images were analyzed using ImageJ (National Institutes of Health). Three regions of interest measuring 150 × 150 µm were taken from random nonoverlapping areas ≥100 µm below the surface in both superficial and growth zones.^10^ To count live and dead cells, a macro was created using the auto local threshold, watershed, and analyze particles functions. A mean was taken from the 3 measured areas to yield a single measurement of viability per sample.

### Histological Evaluation

Samples were fixed in 10% neutral buffered formalin and embedded in paraffin. Cross sections measuring 4 µm in thickness were taken and stained with picrosirius red to assess total collagen distribution and safranin O to assess GAG distribution. Picrosirius red stained samples were additionally viewed under a polarized light to observe orientation of collagen fibers within each sample.

### Quantitative Biochemistry

Samples were weighed 24 hours after steroid exposure to measure wet weights. Samples were then lyophilized for 72 hours and weighed again to measure dry weights. Water content of the explants and constructs was calculated using the measured weights before and after lyophilization. Lyophilized samples were digested in 125 µg/mL papain in phosphate buffer at 60°C for 18 hours. Collagen content was measured using a perchloric acid-free, chloramine-T modified hydroxyproline assay^9^ using Sircol collagen (Biocolor Ltd) as a standard. Sulfated GAG content was measured via Blyscan dimethyl methylene blue assay kit (Biocolor Ltd). A modified assay^5^ was used to quantify hydroxyproline and pyridinoline for degree of crosslinking.

### Mechanical Testing

To provide uniformity, full-thickness, 2 mm–diameter punches were taken from the explant and frozen in protease inhibitor solution until mechanical testing could be performed.^12^ After thawing, a compression apparatus^1^ was used to assess the creep indentation of samples. As previously described,^14^ a 0.88 mm–diameter, flat-ended, porous indenter tip was applied to the samples under a 0.5- to 4.5-g load and allowed to creep until reaching equilibrium. A semianalytical, seminumerical, linear biphasic model and finite element analysis were used to obtain the aggregate modulus and shear modulus of the tested constructs.

For tensile testing, specimens were trimmed into a dog-bone shape as previously described.^38^ Paper tabs were fixed to each end of the sample using a polyurethane adhesive (Gorilla Glue Co), loaded into the grips of a TestResources mechanical tester (TestResources Inc), and pulled at 1% of the gauge length per second until failure. Load changes were recorded throughout testing and used to generate stress-strain curves for the calculation of a Young modulus and ultimate tensile strength for each sample.

### Statistical Analysis

Statistical analysis was performed using GraphPad Prism 9. Sample size (n = 6 per group) was determined based on power analysis from a previous study^21^ using aggregate modulus as the primary outcome with alpha set at .05 and a minimum power of 80%. Statistical outliers were identified via the ROUT method in GraphPad Prism 9 in all data sets and removed before analysis was performed. A 2-way analysis of variance with Tukey post hoc was performed to determine any differences caused by steroid exposure. All data are presented as mean ± SD.

## Results

### Viability and Histology

**Figure 1.**
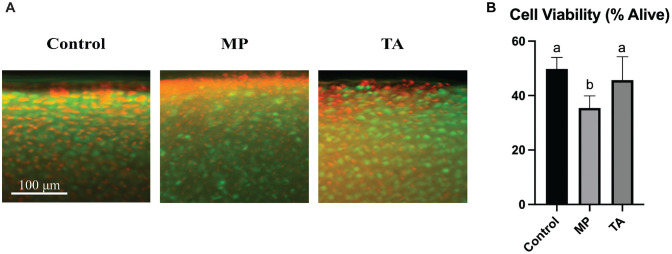
Chondrocyte viability. (A) Surface of explant with overlapping green fluorescent (live) and Texas red (dead) filters at ×20 magnification. (B) A 2-way analysis of variance with Tukey post hoc test was used to determine differences between the 3 groups. Statistical significance is indicated by groups labeled with the letters *a* and *b*. MP, methylprednisolone acetate; TA, triamcinolone acetonide.

**Figure 2.**
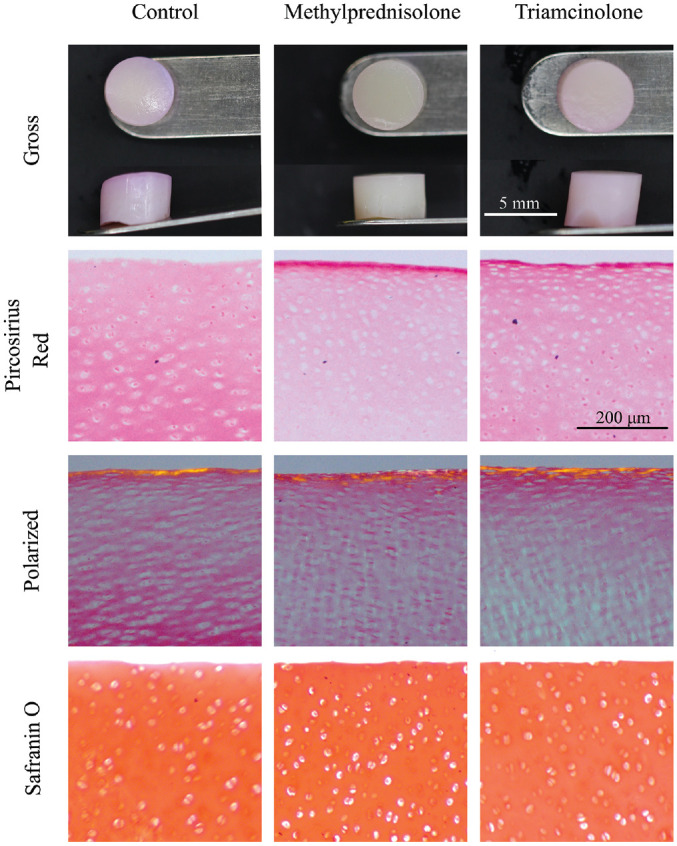
Gross and microscopic histology of native cartilage explants after exposure to either chondrogenic medium (negative control), methylprednisolone acetate, or triamcinolone acetonide.

Chondrocyte viability was decreased in explants exposed to a single dose of MP (35.5%) compared with control (49.8%; *P* < .001) and TA (45.7%; *P* = .01) (Figure 1). No differences in viability were found between control and TA groups (*P* = .570). Histologically, chondrocyte death was concentrated in the superficial zone of the tissue in all groups, with a particular increase in density in the MP-exposed explants. Gross morphology of the explant was unchanged in the steroid-exposed groups compared with control (Figure 2). Collagen distribution appeared similar between all 3 groups, and orientation of collagen fibers examined under polarized light remained unaffected. GAG staining demonstrated equal intensity in all 3 groups.

### Quantitative Biochemistry

No significant differences in collagen and GAG content were found between the 3 groups (Table 1). Calculated percentages of collagen and GAG were consistent with percentages reported in previous studies.^21^ The TA-exposed explants had a significantly lower hydration status compared with control (*P* = .037) and MP-exposed groups (*P* = .029). The degree of collagen crosslinking measured by millimole of pyridinoline per mole of hydroxyproline was significantly lower in the TA-exposed group compared with control (*P* = .027) and MP (*P* = .05) groups. No significant differences were found in degree of crosslinking between the control group and the MP group (*P* = .874).

**Table 1:** Biochemical Composition of the Extracellular Matrix of Articular Cartilage Explants^*a*^

Sample	Hydration, %	Col/WW, %	Col/DW, %	GAG/WW, %	GAG/DW, %	PYR/OHP, mmol/mol
Control	87.3 ± 3.3	8.3 ± 2.8	58.8 ± 1.8	4.2 ± 1.8	27.8 ± 7.8	16.8 ± 1.2
MP	87.5 ± 1.7	8.6 ± 1.7	59.5 ± 5.2	3.7 ± 1.1	25.6 ± 5.4	16.4 ± 1.3
TA	82.9 ± 2.2^*b*^	9.9 ± 1.3	57.8 ± 1.7	4.6 ± 1.7	25.9 ± 6.5	14.4 ± 1.6^*b*^

a Values are expressed as mean ± SD. Col, collagen; DW, dry weight; GAG, glycosaminoglycan; MP, methylprednisolone acetate; OHP, hydroxyproline; PYR, pyridinoline; TA, triamcinolone acetonide; WW, wet weight.

b Value is significantly different from the other 2 groups (*P* < .05).

### Mechanical Properties

**Figure 3.**
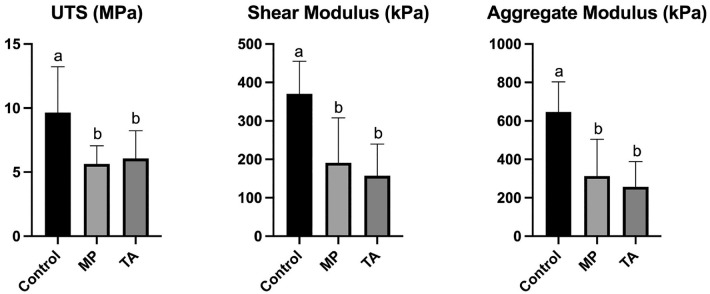
Differences in the mechanical properties of articular cartilage after exposure to steroid. Statistical significance is demonstrated with the letters *a* and *b*. MP, methylprednisolone acetate; TA, triamcinolone acetonide; UTS, ultimate tensile strength.

A significant decrease was seen in the tensile and compressive mechanical properties of native cartilage in steroid-exposed explants when compared with controls, with decreases in ultimate tensile strength (9.650 vs 5.648 MPa [MA; *P* = .021] and 6.065 Mpa [TA; *P* = .0403]), shear modulus (370.1 vs 191.2 kPa [MA] and 157.4 kPa [TA]; *P* < .001), and aggregate modulus (646.3 vs 312.8 kPa [MA] and 257.0 kPa [TA]; *P* < .001) (Figure 3). The Young modulus in TA-exposed native cartilage (7.924 Mpa) was significantly lower than that in MP-exposed explants (12.35 Mpa; *P* = .026) but not significantly different compared with control (11.97 Mpa; *P* = .072).

## Discussion

The results of this in vitro study suggest that a single clinical dose of MP is chondrotoxic and that a single clinical dose of MP or TA can lead to substantial early decreases in both the tensile and the compressive properties of native articular cartilage. Although there were no detectable changes to the extracellular matrix composition or structure, rejecting part of the study's hypothesis, a single 1-hour steroid exposure was enough to cause altered mechanics in native explants 24 hours after exposure, which raises the concern for risk of mechanical failure of the cartilage tissue.

The use of IA corticosteroids for joint pain relief remains controversial for practicing clinicians. IA inflammation can cause significant joint effusion, pain, and stiffness, and corticosteroids are proven mitigators of that inflammation. However, recent data have demonstrated the potential for steroids to be chondrotoxic, calling into question the overall risks of their use in joints with cartilage pathologies.^18,36^ Despite this, the use of IA injections of MP and TA remains a staple in clinical practice, particularly in the treatment of osteoarthritis. Therefore, more data are needed to determine the exact effects of IA steroids across the spectrum of treatment, from a single dose to long-term exposure.

To date, the effects of TA and MP on the mechanical properties of native articular cartilage have not been well studied. Murray et al^19^ examined the effect of 4 IA injections of 40 mg/mL MP on the articular cartilage in an equine model over an 8-week period. The cartilage in the treated joints was tested using creep indentation and was found to be significantly weaker across multiple factors, including aggregate modulus and shear modulus. The current study found similar results after just a single exposure to either MP or TA, demonstrating significantly lower shear and aggregate moduli in the steroid-treated cartilage. Additionally, tensile properties in the steroid-treated explants were significantly decreased, indicating a potential global decrease in mechanical properties after a single exposure to MP or TA.

Changes in the mechanical properties of articular cartilage have been associated with various risk factors for progression of osteoarthritis, including accelerated catabolism,^30^ structural changes,^28^ and impaired cell-to-cell communication.^7^ The shear modulus of steroid-exposed explants in this study decreased 48% to 57% compared with that of controls. In human knee cartilage, decreases in shear modulus of this magnitude have been correlated with the progression from healthy knee cartilage to International Cartilage Regeneration & Joint Preservation Society grade 1-2 osteoarthritis.^23^ Furthermore, decreases in the Young modulus by 25% to 50% have been correlated with the same degree of osteoarthritis,^17^ and decreases of similar magnitude in the Young modulus of TA-exposed cartilage were observed in this study. Even if these effects are only transient, these mechanical changes may predispose the cartilage tissue to initial failure, and correlations of these mechanical changes with an osteoarthritic state are cause for concern for the treating clinician. Future studies examining similar mechanical changes in human articular cartilage after steroid exposure will further aid clinical decision-making, particularly regarding the use of IA steroids in younger patients with healthy articular cartilage.

The mechanical properties of cartilage are often correlated with a variety of biochemical factors within the extracellular matrix, including GAG content,^27^ collagen content,^4^ and degree of pyridinoline crosslinking.^5^ The negatively charged proteoglycan core of GAGs contributes to the overall fixed charge density of cartilage, helping to maintain its compressive stiffness.^20^ Collagen content and its degree of crosslinking via pyridinoline help maintain the integrity of the tensile and shear properties of articular cartilage.^4,11^ Badurski and colleagues^2^ examined the effects of MP on articular cartilage and found dose-dependent decreases in GAG starting at 7 mg in rabbits. Additionally, Sherman et al^29^ found significant decreases in both cartilage and synovial metabolism after MP exposure. Literature on TA has yielded similar results, with a study by Jansen et al^16^ showing that brief exposure to TA can limit GAG synthesis in canine articular chondrocytes and multiple studies demonstrating that TA has the potential to limit collagen synthesis in murine articular cartilage.^35,37^ Despite significant decreases in compressive stiffness of the steroid-exposed cartilage in the current study, no difference in GAG content was found between the control and steroid-exposed groups. One potential explanation for this finding is that changes in GAG content may not be detectable by conventional biochemical assays in the early stages of insult to cartilage.^7^ Additionally, changes in fixed charge density, rather than quantity of GAG, could result in similar changes in compressive properties without detectible changes on conventional biochemical assay.^20^

Similar to GAG, no appreciable differences were found in collagen content despite decreases in tensile properties in the steroid-exposed groups. This change can potentially be explained by changes in collagen crosslinking. Immature divalent crosslinking via dihydroxylysinonorleucine (DHLNL) was not measured in this study and has been shown to contribute substantially to the tensile properties of cartilage.^11^ A decrease in DHLNL would explain the decrease in tensile properties seen in the MP-exposed groups, particularly due to the fact that juvenile bovine cartilage was used in this study and there is potential for a relatively higher concentration of DHLNL to be found in immature tissues.^5^ Therefore, although substantial biochemical changes corresponding with the mechanical changes were not seen in this study, future studies using more exhaustive methods of measuring for fixed charge density and DHLNL may be warranted.

The effects of IA steroids on the viability of cartilage have been a long-standing concern for clinicians. Both basic science studies and clinical trials have found that IA injections of MP and TA can lead to measurable decreases in cartilage volume and chondrocyte viability.^18,19,36^ This study found that only MP was chondrotoxic after a single exposure and that viability in the TA-exposed groups was not significantly different from control. Similar findings were seen in a study by Sherman et al,^29^ who exposed canine articular cartilage to MP and TA for a 24-hour period and found that MP-exposed groups demonstrated a significant decrease in viability whereas TA-exposed groups demonstrated no difference from control. One potential explanation for the difference seen in both the current study and the study by Sherman et al from the conventional literature is that the steroid exposures were short-term, and the chondrotoxic effect of IA steroids has proven to be both concentration- and time-dependent.^36^ The effect of longer or repeated exposures of MP and TA to articular cartilage is therefore an important consideration deserving of further study.

The results of this study must be taken within the context of its limitations. First, a 1-hour exposure of steroid may not be an accurate representation of the natural course of MP and TA in the joint, as traces of these steroids have been found in the synovial fluid for up to 14 days after IA injection.^6^ Future studies can add to the validity and clinical understanding of these results by examining the effects of long-term or recurring steroid exposure on fresh, human articular cartilage. Second, the cartilage tissue used in this study was juvenile bovine explant cartilage, not human. The harvesting of explants from bovine specimens 36 hours after slaughter, as well as the stresses from transition to in vitro culture, inevitably led to some cell death. Additionally, compared with mature adult cartilage, juvenile articular cartilage is much more cellular and metabolically active, and these stressors may have led to increased cell turnover as detected on the live/dead assay. Although these factors partially explain the relatively lower viability in the control specimens, 50% viability is still lower than desired for control specimens, and the detrimental effects observed in this study need to be confirmed on mature human cartilage tissue. Third, corticosteroids are often injected in conjunction with local anesthetics, whereas this study focused on the toxicity of MP and TA alone. However, other studies examined the intra-articular effects of local anesthetics and reported similar findings of dose-dependent chondrotoxicity and mechanical weakening.^21,29^ Finally, recent studies have raised concern over the effect of the freeze-thaw cycle on the mechanics of native cartilage.^24^ The cartilage explants used in this study were stored at –20°C and underwent 1 freeze-thaw cycle, whereas changes in compressive mechanical properties have only been observed in specimens that undergo freeze-thaw cycles at temperatures –80°C or colder.^32^ Therefore, –20°C storage is unlikely to affect the compressive stiffness or structural integrity of the collagen fibrils of the explants in this study.

## Conclusion

This study found that a single 1-hour exposure of MP to native articular cartilage explants was significantly more chondrotoxic than TA or control. Additionally, a single 1-hour exposure of MP or TA to native articular cartilage explants resulted in significant decreases in both compressive and tensile mechanical properties of the cartilage. Clinicians should be judicious regarding use of IA steroids, particularly in patients with intact healthy articular cartilage.
